# Discrete Pattern of Burst Stimulation in the Ventrobasal Thalamus for Anti-Nociception

**DOI:** 10.1371/journal.pone.0067655

**Published:** 2013-06-24

**Authors:** Yeowool Huh, Jeiwon Cho

**Affiliations:** 1 Center for Neural Science, Korea Institute of Science and Technology, Seoul, Korea; 2 Department of Neuroscience, University of Science and Technology, Daejeon, Korea; University of North Dakota, United States of America

## Abstract

The thalamus has been proposed to play a role in sensory modulation via switching between tonic and burst dual firing of individual neurons. Of the two firing modes, altered burst firing has been repeatedly implicated with pathological pain conditions, which suggests that maintaining a certain form of thalamic burst could be crucial for controlling pain. However, specific elements of burst firing that may contribute to pain control have not yet been actively investigated. Utilizing the deep brain stimulation (DBS) technique, we explored the effects of bursting properties in pain control by electrically stimulating the ventrobasal (VB) thalamus in forms of burst patterned to test different aspects of bursts during the formalin induced nociception in mice. Our results demonstrated that electrical stimulations mimicking specific burst firing properties are important in producing an anti-nociceptive effect and found that the ≤3 ms interval between burst pluses (intra-burst-interval: IntraBI) and ≥3 pulses per burst were required to reliably reduce formalin induced nociceptive responses in mice. Periodicity of IntraBI was also suggested to contribute to anti-nociception to a limited extent.

## Introduction

Deep brain stimulation (DBS) has been widely used to treat many different types of pathological conditions ranging from *Parkinson’s disease* to *depression* [1]. Pathological pain conditions also have been attempted to be treated with DBS with limited successes and the effectiveness of pain relief by the stimulation varied among individuals [2–4]. This may be, in part, due to the stimulation protocol, for example, typical DBS protocols used for pain treatment were continuous stimulations with ≥100 Hz frequency which have weak resemblance to the naturally occurring thalamic discharges. Therefore, remodeling the DBS protocol to have closer resemblance to the natural neuronal signals may endow DBS to enhance therapeutic effects while reducing the side effects.

Among the various brain regions that have been targeted for the treatments of different types of pain conditions, the somatosensory thalamus has often been targeted to treat neuropathic pain for its relay function [2,3,5–7]. The somatosensory thalamus is an intermediary structure which relays peripheral sensory information to the sensory cortex [8]. Due to its strategic position between the periphery and the cortex, the thalamus has long been proposed to modulate peripheral sensory information before transmission to the cortex, thereby, serving a sensory gating role [9,10]. Since a single thalamocortical (TC) neuron is able to fire in single spikes (tonic firing) or in a burst of high frequency spikes (burst firing) via its mutual connections between the cortex and the reticular thalamus (RT) [11–13], the sensory gating role is thought to occur by switching between the two firing modes. The two firing modes are considered to have differential roles, for example, tonic firing was observed to be predominant over burst firing in the awake state while burst firing became more frequent during sleep [14–17], hence burst firing was initially considered to block transmission of sensory information. However, since burst firing frequency was reported to be elevated during the awake state of patients suffering from pain, its prevalence in the awake state was deemed to be a pathological condition causing the pain experience [18,19]. However, animal studies revealed that burst firing properties have been altered in neuropathic pain models compared to those of the intact animals [20], alluding that burst firing in pain patients could have been altered into a form that would serve completely different functions from that of non-neuropathic pain transmission mechanisms. Furthermore, a recent study has shown that only specific forms of burst had an anti-nociceptive effect [21], implicating the important role of bursting patterns in producing anti-nociception. Based on previous findings, we sought to further investigate the components of a burst which could possibly contribute to the anti-nociceptive experience by measuring the differential anti-nociceptive effects of different DBS stimulation protocols in forms of bursts. For this, the formalin test was used because it produces tonic pain, which was suggested to have close resemblance to most clinical pain [22].

Of the many thalamic nuclei, the ventrobasal (VB) complex, which includes the ventro-posterior lateral (VPL) and the ventro-posterior medial (VPM) nuclei, was targeted for the stimulation, since it is suggested to serve a sensory gating function in rodents.

The present study investigated the effect of burst components by stimulating the VB with varying burst protocols to compare the nociceptive responses induced by formalin based on the hypothesis that bursts with different properties would have different impacts on anti-nociception. Consistent with our hypothesis, we found that the number of burst pulses composing a burst, the intra-burst-interval (IntraBI), and the possible influence of periodicity of IntraBI play important roles in reducing nociceptive responses.

## Results

### The effect of electrode implantation in the VB

**Figure 1:**
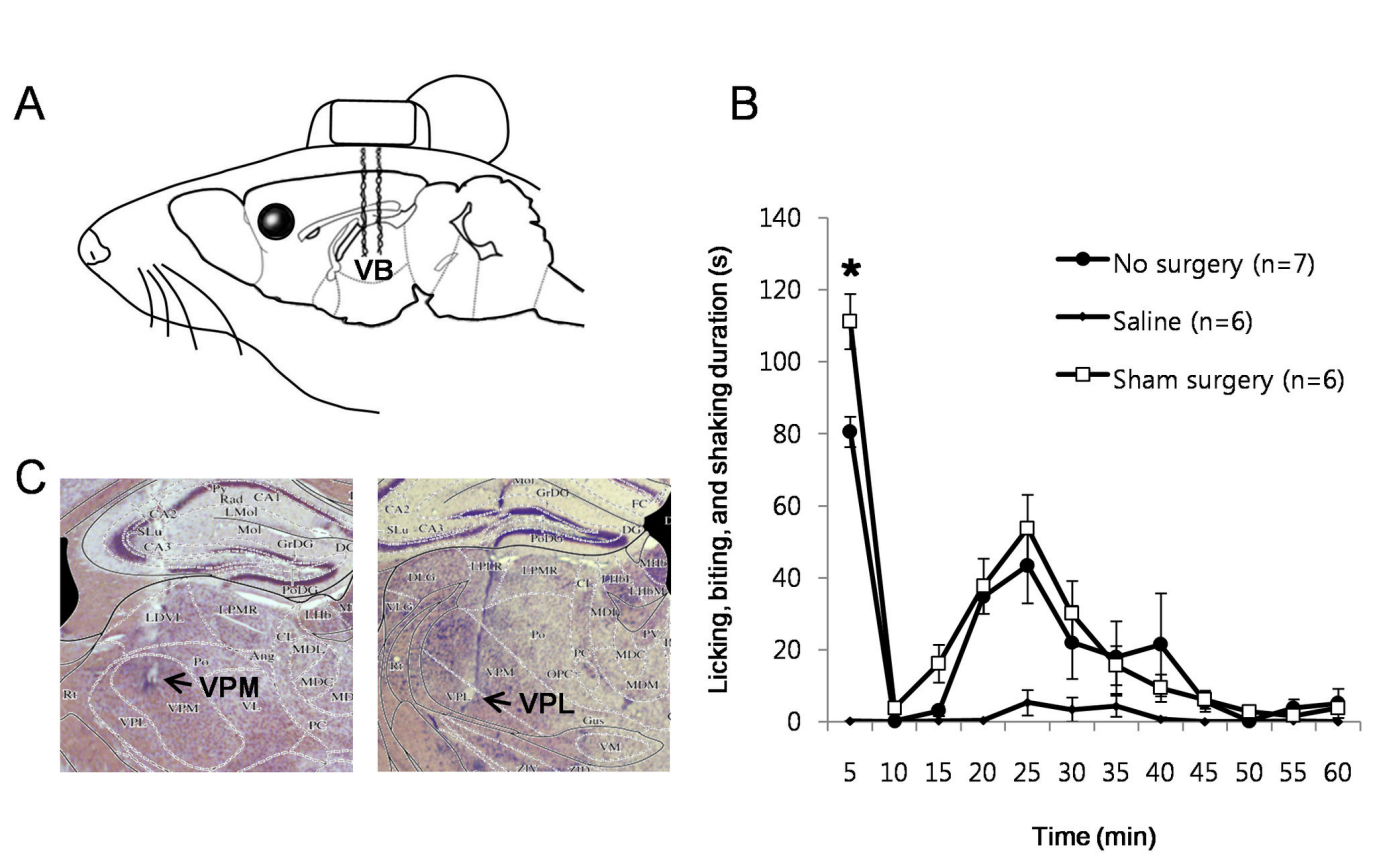
Stimulating electrode implant and anti-nociception. (A) Schematic drawing of the bipolar stimulating electrode aligned in the VB. (B) Influence of stimulating electrodes on formalin induced behavioral nociception. No surgery: no surgery + formalin. Saline group: no surgery + saline. Sham surgery: bipolar electrodes implantation surgery + formalin. All data points are mean±SEM. Student’s t-test was used to compare each data point in the No surgery group with respective data points in the sham surgery group, *P<0.05. (C) Histology of the stimulation sites.

Implantation of electrodes for DBS may alter the physiological state of the brain through the electrode-brain interface [23,24] that may ultimately cause changes in the behavioral responses to a nociceptive stimulus. A clinical study reported that implantation of electrodes in the somatosensory thalamus relieved neuropathic pain symptoms in some patients even before any stimulation was given [25], eluding that implantation of electrodes itself could have a therapeutic effect. We therefore investigated whether electrode implantation in the VB of mice would have an anti-nociceptive effect to formalin induced nociception by comparing differences in the nociceptive response between the sham surgery and the ‘no-surgery group’. The sham surgery group went through an electrode implant surgery in the VB and had stimulation cords attached to the module with electrodes during the formalin test without actual electrical stimulations being delivered. The ‘no-surgery group’ had the formalin test without any surgical operations. A schematic drawing of electrode implantation configuration is shown in Figure 1A and histological samples of electrode implantation sites are shown in Figure 1C.

Formalin injection (5%, 10 µl) to the hind paw pad of a mouse triggered a biphasic surge of behavioral nociceptive responses, a typical response pattern in the formalin test, in both the sham surgery and ‘no-surgery group’ (Figure 1B). The level of the 1^st^ phase (0-5 min) nociceptive responses was higher for the sham surgery group, while the 2^nd^ phase (10-60 min) nociceptive responses of the two groups did not differ. Contrary to the clinical result, implantation of electrodes in the VB did not reduce the degree of formalin induced nociceptive responses in mice, but rather increased nociceptive responses only in the 1^st^ phase. The discrepant results may be due to the different nature of pain—neuropathic vs. nociceptive pain of the 1^st^ phase—or placebo effects in human patients. The results also indicate that the stimulating cord attached to a mouse did not interfere with the expression of nociception related behaviors.

### Effects of stimulations with various Intra-burst-intervals

We further tested which component or components of the burst stimulation could be contributing to the anti-nociceptive effect in the VB. A recent study from our lab showed that increased occurrence of bursts in the VB was tightly correlated to the reduction of nociceptive responses and further demonstrated that bursts could indeed reduce nociceptive responses using an electrical stimulation mimicking thalamic bursts [21]. The same study also alluded that not all forms of bursts would equally contribute to reduce nociceptive responses, suggesting that specific bursting properties would be essential for bursts to have an anti-nociceptive effect. Since a thalamic burst is composed of a pre-burst hyperpolarization, an IntraBI, and at least two burst spikes, we attempted to find the burst component that is essential for the anti-nociceptive effect by modifying the IntraBI and the number of burst pulses in the following stimulation tests. The effect of pre-burst hyperpolarization could not be tested since it cannot be controlled by means of electrical stimulations.

The first bursting component tested was the length of IntraBI. Since the length of IntraBI had a tendency to be shortened while nociceptive responses were relieved in our previous study [21], the precise arrangement of IntraBI was suggested to be important for pain control.

**Figure 2:**
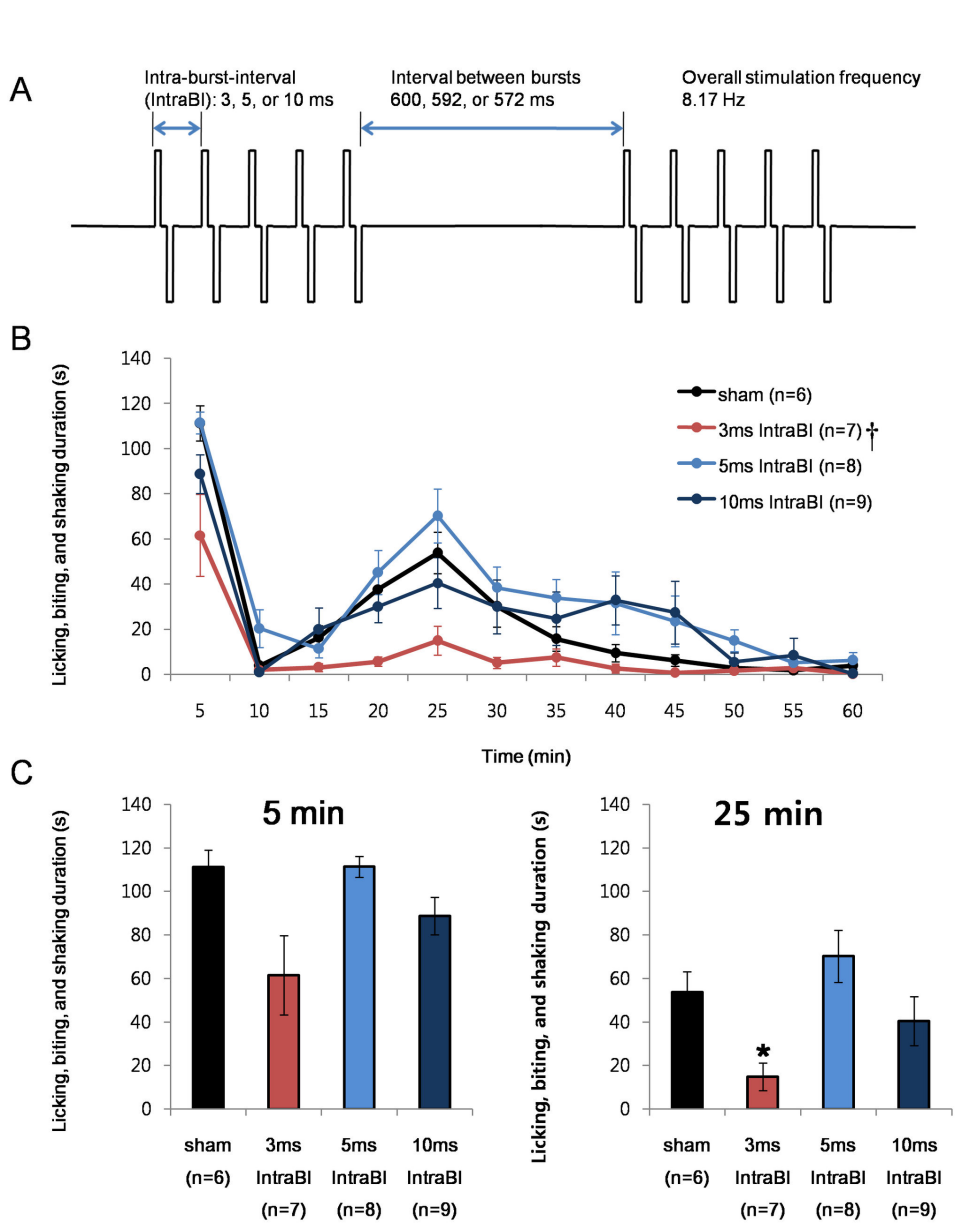
IntraBI and anti-nociception. (A) Schematic drawing of electrical stimulation protocols. (B) Comparison of the effect of VB stimulation with varying IntraBI length on formalin induced nociceptive responses. Repeated measures ANOVA was used for statistical analysis over time followed by Games-Howell post hoc. †P<0.05 (C) Bar graph of the time segments representing the peak of the 1^st^ (0-5 min; F=4.58, P<0.05) and 2^nd^ (20-25 min; F=4.90, P<0.05) phase nociceptive responses for better comparisons between different stimulation conditions. All data points are mean±SEM. One-way ANOVA followed by Games-Howell post hoc was used to compare each data point with the sham control, *P<0.05.

The nociceptive responses of 3 stimulating conditions with the 3, 5, and 10 ms IntraBI were compared. All groups were stimulated with 5 pairs of bipolar square pulses (100 µs width and 100 µA intensity) as delineated in the schematic drawing (Figure 2A). Bipolar stimulation was intended to localize the stimulation to the VB region and minimize the spread of electrical currents to adjacent brain areas that do not have direct connection with the VB; nonetheless, due to the nature of electrical stimulation, structures connecting to the VB would also be stimulated. The width and intensity of the stimulating pulse were chosen to be within the parameters often used for several DBS studies [26]. The interval between bursts were set to be approximately 600 ms to mimic the average value of the silent periods before a thalamic burst during the time segment of reduced nociceptive responses [21]. The overall stimulating frequency was also fixed to be approximately 8 Hz by modifying the interval between bursts, 600 ms, 592 ms, and 572 ms respectively for the 3 ms, 5 ms, and 10 ms IntraBI stimulations.

The results show that only the 3 ms IntraBI stimulation effectively reduced the nociceptive responses in the 2^nd^ phase 15-20 min (F=5.07, P<0.01; Games-Howell post hoc P<0.05) and 20-25 min (F=4.90, P<0.01; Games-Howell post hoc P<0.05) segments (Figure 2B). The 0-5 min and 20-25 min segments after formalin injection, respectively corresponding to the peak of nociceptive responses of each phase, are delineated in bar graphs for better comparison between groups (Figure 2C). Only the stimulating condition that was within the IntraBI range for low threshold Ca^2+^ spike (LTS) burst (≤4 ms IntraBI [27]) yielded an anti-nociceptive effect. In addition, since pain responses for the 1^st^ and 2^nd^ phases are known to occur through different mechanisms, i.e., the acute responses due to the direct activation of nociceptors during the 1^st^ phase and gradual and long-lasting inclination and declination of nociceptive responses due to the development of inflammation during the 2^nd^ phase [28,29], the 3 ms IntraBI stimulation appears to be more effective in reducing the 2^nd^ phase nociceptive responses.

### Effects of burst pulses number

**Figure 3:**
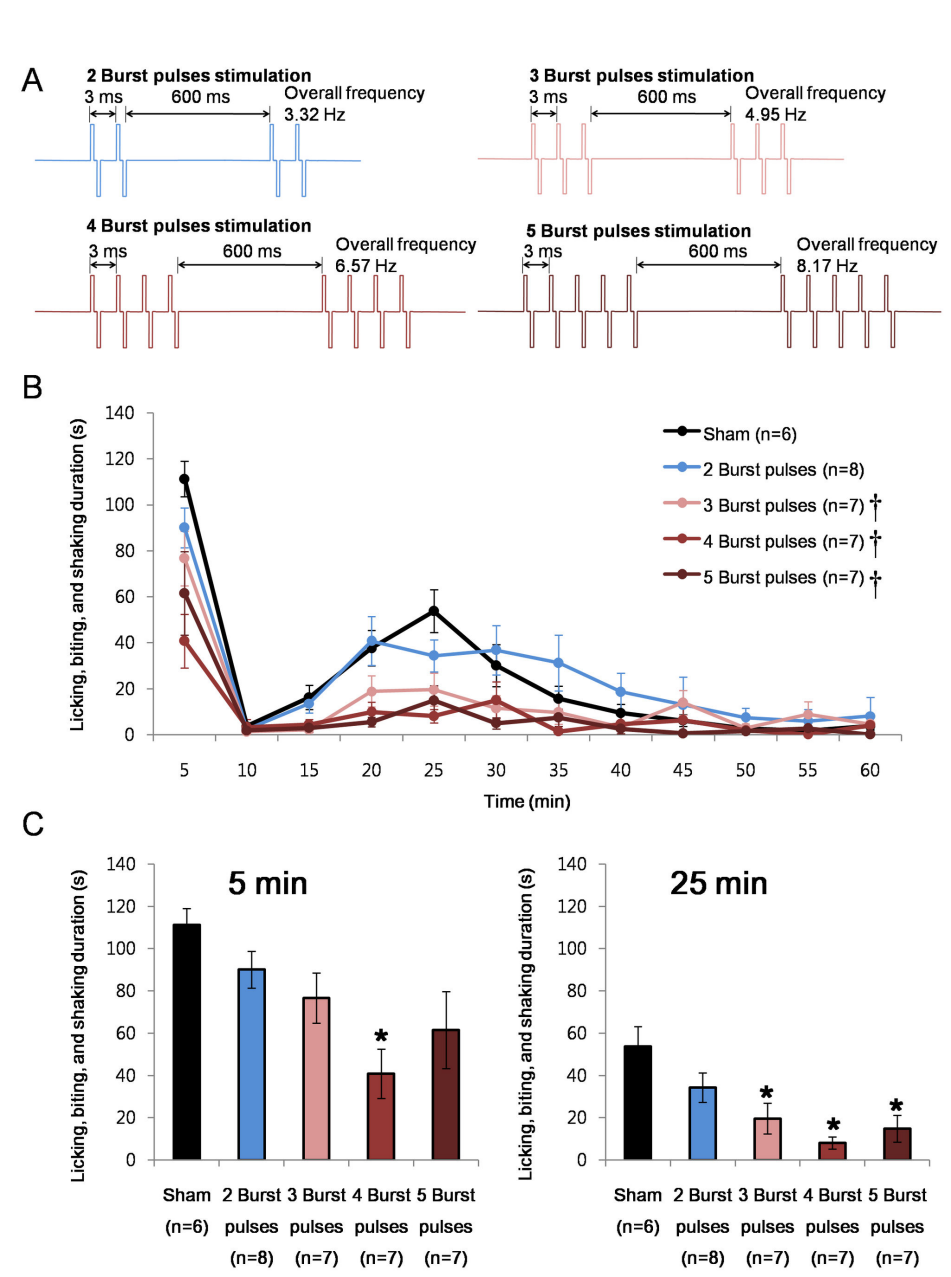
Number of burst pulses within a burst and anti-nociception. (A) Schematic drawing of electrical stimulation protocols. (B) Comparison of the effect of VB stimulation with different burst pulse number per burst on formalin induced nociceptive responses. Repeated measures ANOVA was used for statistical analysis over time followed by Games-Howell post hoc. †P<0.05 (C) Bar graph of the time segments representing the peak of the 1^st^ (0-5 min; F=4.56, P<0.05) and 2^nd^ (20-25 min; F=6.74, P<0.05) phase nociceptive responses for better comparisons between different stimulation conditions. All data points are mean±SEM. One-way ANOVA followed by Games-Howell post hoc was used to compare each data point with the sham control, *P<0.05.

A previous study has shown that the number of burst spikes had a tendency to increase when nociceptive responses were significantly reduced during the 2^nd^ phase [21], suggesting that the number of spikes within a burst may also have an important role in reducing nociception. Its significance was verified using electrical burst stimulations with varying numbers of burst pulses. All groups were stimulated with bipolar pulses with 100 µs width and 100 µA intensity. Since 3 ms was the only IntraBI effective in reducing nociceptive responses, all groups were designed to have 3 ms IntraBI with a 600 ms inter burst interval (Figure 3A).

Results showed that the number of pulses within a burst also plays a critical role in reducing nociceptive responses. Three or more burst pulses were required to significantly reduce the nociceptive responses in the 2^nd^ phase while only the 4 burst pulse stimulation significantly reduced nociception in the 1^st^ phase (Figure 3). The effective range of anti-nociception in the 2^nd^ phase was slightly different for different stimulation groups. Stimulations with greater number of burst pulses had a significant tendency to have a longer anti-nociceptive range: 15-20 min (F=4.01, P<0.005; Games-Howell post hoc P<0.05) and 20-25 min (F=6.74, P<0.001; Games-Howell post hoc P<0.05) segments under the 5 burst pulse stimulation, and 20-25 min (F=6.74, P<0.001; Games-Howell post hoc P<0.05) segment under the 4 and 3 burst pulse stimulations. The 2 burst pulse stimulation showed no anti-nociception during the entire recording period. Figure 3B illustrates these changes over time while Figure 3C shows the nociceptive responses of 0-5 min and 20-25 min segments, each representing peak responses of each phase for better comparison between groups.

### Effect of IntraBI periodicity

**Figure 4:**
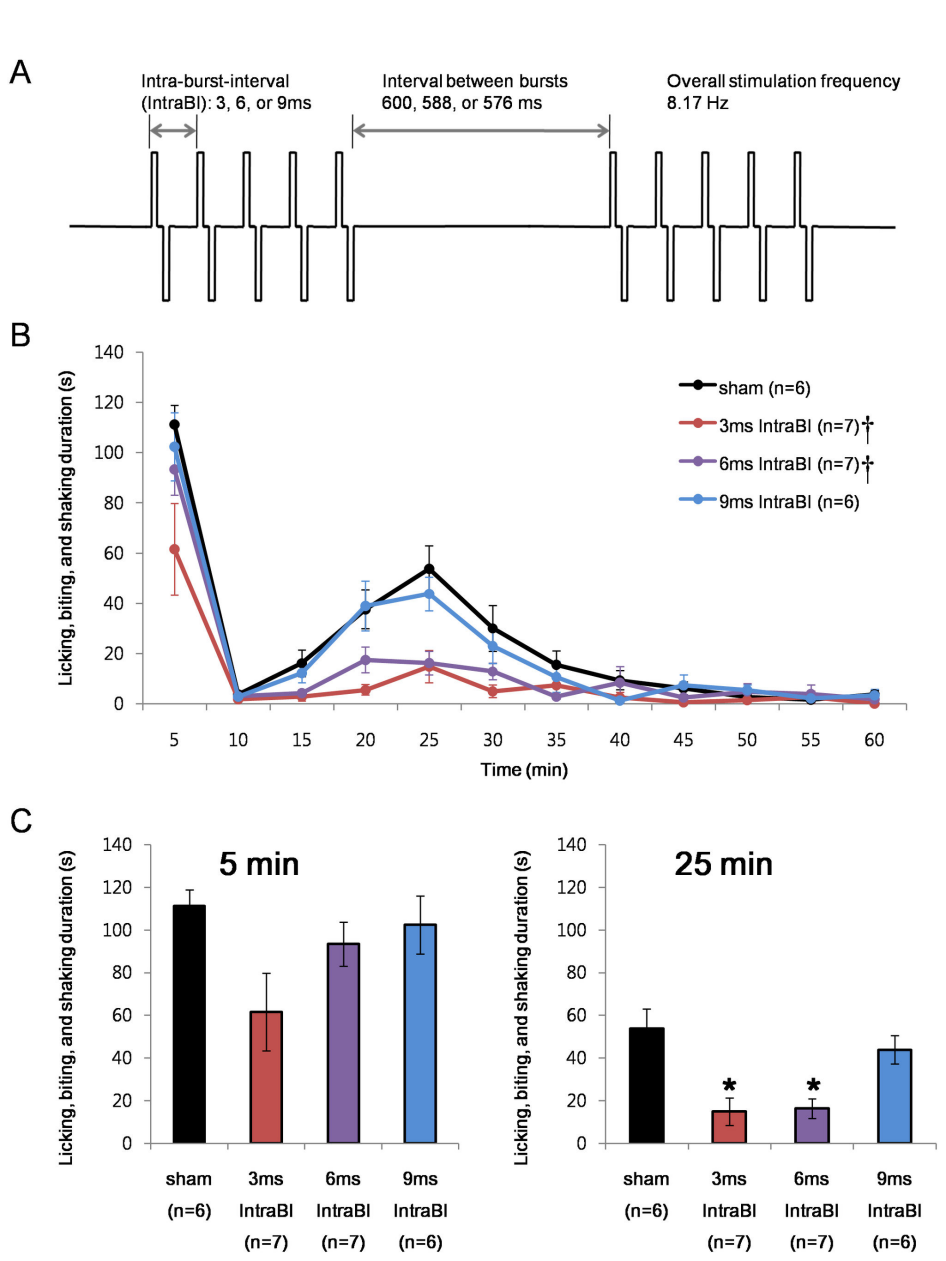
Within burst periodicity and anti-nociception. (A) Schematic drawing of electrical stimulation protocols. (B) Comparison of the effect of VB stimulation with IntraBI in multiple of three on formalin induced nociceptive responses. Repeated measures ANOVA was used for statistical analysis over time followed by Games-Howell post hoc. †P<0.05 (C) Bar graph of the time segments representing the peak of the 1^st^ (0-5 min; F=2.67, P=0.07) and 2^nd^ (20-25 min; F=8.35, P<0.05) phase nociceptive responses for better comparisons between different stimulation conditions. All data points are mean±SEM. One-way ANOVA followed by Games-Howell post hoc was used to compare each data point with the sham control, *P<0.05.

During sleep, thalamocortical cells burst rhythmically, and the rhythmical bursting is hypothesized to block sensory information from being transferred to the cortex [16]. Likewise, the within burst periodicity may have been the key factor in reducing nociceptive responses. Therefore, stimulations set to have IntraBIs with a multiple of 3 were tested, e.g., 6 ms and 9 ms IntraBIs. The three stimulation conditions had the identical pulse width and duration, number of pulses within a burst, and overall stimulating frequency as described in Figure 4A. The overall stimulation frequency was kept constant by modifying the interval between burst stimulations to 600 ms, 588 ms, and 576 ms for 3 ms, 6 ms, and 9 ms IntraBI stimulations, respectively. The stimulation with IntraBI of 6 ms produced a similar level of nociceptive pain responses to that of the sham controls in the 1^st^ phase (0-5 min after formalin injection) while nociception was significantly reduced in the 2^nd^ phase (20-25 min after formalin injection). The 9 ms IntraBI stimulation, on the other hand, could not reduce nociceptive responses in both phases.

It is noteworthy to mention that the 6 ms IntraBI stimulation reduced the 2^nd^ phase nociceptive responses while the 5 ms IntraBI was ineffective in reducing them. This suggests that periodicity of IntraBI in multiples of 3 may influence the effect of burst stimulations in reducing nociceptive responses, although it appears to be only effective at a very short range of IntraBI.

## Discussion

This study reports that certain bursting properties are critical for endowing electrical stimulations with its anti-nociceptive ability. Of the parameters tested, both IntraBI and the number of burst pulses were determined to be critical factors in producing anti-nociceptive effect. IntraBI of ≤3 ms and burst pulses of ≥3 were required to reliably reduce formalin induced nociceptive responses. On the other hand, the periodicity of IntraBI, in a multiple of 3, had limited effects on anti-nociception in the 2^nd^ phase responses when the IntraBI was relatively short (6 ms).

The burst stimulation patterns tested in our investigation were modeled based on the previous neuronal recording data of VB neurons during the formalin test to isolate the elements of bursting properties that were required for anti-nociception. Considering the nature of the electrical stimulation method, the temporal patterning of burst stimulation would act at multiple levels of the TC circuitry to produce an anti-nociceptive effect. First of all, the RT, which provides the major source of GABAergic inhibition to the TC neurons to initiate LTS bursting in the thalamus [11], could have been preferentially activated by the TC neuron stimulation to generate more bursts in the VB in return since TC input to the RT above a certain threshold was shown to trigger regenerative generation of bursts in the thalamus [30]. The regenerative LTS burst production, accompanied by intrinsic hyperpolarization, could lead the VB to greater depression of tonic firing and have consequently reduced the expression of nociceptive pain at the thalamic level. Our previous paper that investigated the respective roles of tonic and burst firing in nociceptive signaling have also suggested that increasing burst firing in the VB initiated reduction in tonic firing and overall firing rate, which eventually led to the reduction in nociceptive responses [21], by increasing the occurrence of bursts and the pre- & post-hyperpolarizations accompanied by bursts [21].

The cortex, another structure connected with the TC neurons [8], could also have been affected by the stimulation to produce the anti-nociceptive effect. Stimulating the VB neurons with a specific bursting pattern may have greater potency to activate the inhibitory interneurons over the excitatory ones in the cortex, since bursting of TC neurons were suggested to have greater potency to activate the cortical inhibitory interneurons over the excitatory ones [31,32]. Therefore, increasing inhibition in the cortical layer by specific burst stimulations could have blocked nociceptive pain signal transmission, eventually leading to the reduction in nociceptive responses.

Anti-dromic activation of the spinal cord neurons could also have produced the anti-nociceptive effect. High frequency burst stimulations in the spinal cord have been reported to be more effective in controlling pain with reduced side-effects than the conventionally used continuous high frequency stimulation [33]. However, unlike our study which used approximately 8 Hz overall stimulating frequency at maximum, the spinal cord stimulation study used an extremely high stimulation frequency (200 Hz overall; 40 Hz between bursts with 5 pulses at 500 Hz per burst). Therefore, the anti-nociception shown in our study is unlikely to be caused by anti-dromic stimulation of the spinal cord since the overall stimulation frequency was relatively low at approximately 8 Hz or lower overall, 1.63 Hz between bursts and 333 Hz within a burst.

Activation of other somatic sensations and symptoms such as paresthesia may be another possible factor contributing to the reduction of nociceptive responses due to the competition. The VB relays not only nociceptive information, but also other sensations such as touch or temperature [8]. Therefore, stimulating the VB could have activated sensations other than nociception and consequently had reduced nociceptive signal transmission or had produced paresthesia which in turn could have interfered with the expression of nociceptive responses. Whether mice undergo paresthesia could not be measured but no visible discomfort nor any abnormal behaviors were observed in any mice. In another study, a high frequency (50 Hz) stimulation protocol of DBS in the VB was reported to accompany limbic seizure symptoms such as wet dog shake, head bobbing or rearing behaviors in rodents [34], but no such symptoms were observed in the present study, possibly due to the differences of DBS protocols. Paresthesia is a common side-effect of DBS experienced by human patients [2,35,36], but since the spinal stimulation in the form of bursts reduced paresthesia in human patients [33], it is possible that stimulation in the form of bursts may reduce paresthesia symptoms in general.

The periodicity within a burst may be another factor contributing to anti-nociception to a certain degree, since slight nociceptive effect was present in 2^nd^ phase by the 6 ms IntraBI stimulation whereas the 5 ms IntraBI had no anti-nociceptive effect at all. Differences between the 5 and 6 ms IntraBI stimulations suggest that in addition to the RT-TC contribution, another mechanism, such as generation of anti-nociceptive brain rhythms or another pathway, may have also contributed to produce the anti-nociception effect. Brain rhythms of patients with chronic pain were altered, and the altered brain rhythms are postulated to contribute to the abnormal pain experience of these patients [37]. Direct connection between periodicity of IntraBI and brain rhythm generation may be hard to demonstrate at the moment, but a certain IntraBI may be more effective in generating brain rhythm for anti-nociception. However, since the 9 ms IntraBI stimulation had no anti-nociceptive effect at all, periodicity of IntraBI appears to have only limited effect on anti-nociception.

Even in the burst protocols that have anti-nociceptive effects, the degree of anti-nociceptive effect differed between the 1^st^ and 2^nd^ phases. Only a tendency of nociceptive responses reduction was present in the 1^st^ phase while the 2^nd^ phase nociceptive response was reduced almost completely. This may be due to the different nature of nociceptive pain generation. The 1^st^ phase responses are due to direct activation of nociceptors while the 2^nd^ phase responses are due to the development of inflammation by nociceptive stimuli [28,29,38]. Previous studies have shown that bursts would be involved in reducing pain only in the long-term pain models where nociceptive responses persists for several minutes or longer [21,39]. Therefore, the burst stimulation may be more effective in reducing the 2^nd^ phase responses rather than the 1^st^ phase responses.

In general, our study demonstrated that burst stimulation with shorter IntraBI, greater number of burst pulses and relatively long intervals between bursts could successfully reduce formalin induced nociceptive responses. This result is consistent with the previous study reporting that bursts analyzed from recordings of spontaneous firing of neuropathic animals had reduced the number of burst spikes and increased IntraBI compared to the intact control animals. Sometimes increased bursting in the thalamus during the awake state is assumed to be a factor causing neuropathic pain [18,19]. However, if not all forms of burst firing contribute equally to the pain experience, different forms of burst could have differential effects on the nociceptive pain experience. Indeed, continuous high frequency electrical stimulation (>100 Hz) in the sensory thalamus was shown to reverse the altered burst firing properties of neuropathic pain model to near normal [40]. In other words, bursts with relatively longer IntraBI and less burst spike number could possibly increase nociceptive responses while the opposite form of burst would have the contrary effect.

Overall, this study showed that electrical burst stimulations with specific properties would have different anti-nociceptive effects. Future investigation utilizing optogenetic tools would help dissociate the precise circuitry and action mechanism of burst stimulations on pain control.

## Materials and Methods

### Ethics statement

All experiments were conducted in accordance to the guidelines of the Committee for Research and Ethical Issues of International Association for the Study of Pain [41] and approved by the Animal Care and Use Committee of Korean Institute of Science and Technology (protocol number: AP-2011L7006). Implant surgeries were done under urethane anesthesia and all efforts were made to minimize suffering of animals during the experiment.

### Subjects

First generation of C57BL/6J × 129/SvJae hybrid mice (male 10-14weeks, body weight 25-30 g) were used in the experiment. C57BL/6J × 129/SvJae hybrids were bred in the animal facility in Korea Institute of Science and Technology. Both inbred mouse lines, C57BL/6J and 129/SvJae, were obtained from The Jackson Laboratory (USA). Mice were maintained at constant temperature (22±1°C) with free access to food and water under a 12 h light and dark cycle (light cycle beginning at 8: 00 AM).

### Behavioral assessment of nociceptive responses

The formalin test was used to induce nociceptive pain. All mice used in the experiments were handled and habituated to the experimental setting for a week for approximately 10 min including the test day. The test chamber was an opaque plastic cylinder (diameter: 20 cm, height: 25 cm) placed on top of a beveled mirror for behavior monitoring. Either saline (0.9%) or 10 µl of 5% formalin (1:20 dilution of 37% formalin solution in double de-ionized H_2_O) was injected subcutaneously into the plantar surface of the left hind paw with a syringe (Hamilton, USA). Immediately after the injection, behavior was videotaped with a camcorder (SONY HDR-SR11, Japan) for an hour and manually analyzed by two blinded investigators. Nociceptive responses were scored by measuring the total duration of licking, biting, and shaking of the formalin injected paw in 5 min blocks. The scores of the two investigators were averaged.

### Implantation of stimulation electrodes

Mice were anesthetized with zoletil (30 mg/kg i.p.) and sufficient level of anesthesia was maintained throughout the surgery. Surgery was done using a stereotaxic instrument (David Kopf Instruments, USA). Two bipolar stimulating electrodes aligned to be approximately 0.6 mm apart (Teflon-coated stainless steel, 0.003” bare 0.055” coated, A-M Systems, USA) were implanted in the right VB region (VPL and VPM; AP: -1.34, ML: -1.8, DV: -3.2 relative to the bregma [42]) of the brain, which is contralateral to the formalin injection site, and secured onto the skull with a stainless steel screw and dental cement. Mice were handled daily during a week recovery period.

### Electrical stimulation of the ventrobasal thalamus

Different burst-stimulation protocol was given to stimulate the VB thalamic neurons during the formalin test. None of the stimulation conditions caused visible aversion, irritation, or aberrant behavior. All stimulating pulses were biphasic square pulses with current amplitude of 100 µA and duration of 100 µs. Stimulating conditions differed in IntraBI intervals (3, 5, 6, 9 or 10 ms), the number of burst pulses (2, 3, 4 or 5 burst pulses per burst), or interval between bursts (572, 576, 588, 592 or 600 ms). For details of the stimulation protocols refer to Figures 2A, 3A and 4A.

### Statistical analysis

Repeated measures ANOVA was used for statistical analysis over time between experimental groups followed by Games-Howell post hoc test. To analyze the effect of stimulation conditions at different nociceptive phase, statistical analysis was done for each 5 min time segments. First, Levene’s test of equality of error variance was used to determine equal variance at P<0.05. For time segments with equal variance, one-way ANOVA was used while univariate general linear mean was used for time segments with unequal variance, both followed by Games-Howell post hoc test to compare the effect of each stimulation condition to the sham control. Since all the 0-5 min and 20-25 min segments were determined to have equal variance, one-way ANOVA was used for those segments. Student’s t-test was used to compare differences for each 5 min time segments between the sham and ‘no-surgery group’ because main purpose was to compare the difference between the two groups. P-value of 0.05 was used to determine the significance for all statistical analyses. All statistical analyses were done using SPSS (13.0).

### Histology

Stimulation sites were verified post mortem. Mice were overdosed with 2% avertin, and perfused transcardially first with saline and then with 10% formalin (1:10 dilution of 37% formalin solution in 0.9% saline). Brains were removed and further fixed in 10% formalin (1:10 dilution of 37% formalin solution in ddH_2_O) for a day and stored in 30% sucrose solution at 4°C in the refrigerator for a week before sectioning. Coronal sections (50 µm) were cut through the entire thalamus with a microtome cryostat (Microm, Germany). The sections were stained with Cresyl Violet (Sigma, USA) and examined under a light microscope to identify the stimulation sites.
